# Evaluation of the Usefulness of Machine Learning and Artificial Intelligence on Hyperspectral Images in the Diagnosis of Myelodysplastic Syndrome

**DOI:** 10.1002/jbio.70319

**Published:** 2026-07-06

**Authors:** Yasuo Ueda, Takafumi Ogawa, Akane Wada, Genshu Tate, Yuta Baba, Tetsuya Fukuda, Toshiko Yamochi

**Affiliations:** ^1^ Pathology and Laboratory Medicine Showa Medical University Fujigaoka Hospital Yokohama Kanagawa Japan; ^2^ Internal Medicine (Hematology) Showa Medical University Fujigaoka Hospital Yokohama Kanagawa Japan; ^3^ Department of Diagnostic Pathology Showa Medical University School of Medicine Shinagawa Tokyo Japan

**Keywords:** artificial intelligence, bone marrow specimens, hematoxylin and eosin specimens, hyperspectral imaging, myelodysplastic syndrome

## Abstract

Hyperspectral imaging (HSI) has gained increasing use in pathological diagnosis in recent years. HSI captures spectral information at wavelengths beyond the visible range. We analyzed specimens from 36 cases diagnosed with myelodysplastic syndrome (MDS) based on bone marrow biopsy or clot specimens at Showa Medical University Fujigaoka Hospital and classified them into three groups based on dysplastic lineage. HSIs of the biopsy specimens were acquired using a pushbroom hyperspectral camera. Spectral data were extracted from annotated target cells and used to train machine learning classifiers. Pixel‐level data were divided into training and evaluation sets at the sample level and cross‐validated. Our HSI‐based artificial intelligence system achieved high accuracy (up to 97%) in bone marrow pathology, outperforming previous RGB‐based studies and demonstrating the feasibility of HSI for MDS diagnosis. To the best of our knowledge, this study is the first such evaluation on bone marrow specimens.

## Graphical abstract

Hyperspectral imaging (350–1050 nm) captures spectral information beyond the visible range from bone marrow biopsy specimens. An HSI‐based AI system using machine learning achieved up to 97% accuracy in diagnosing and classifying MDS, outperforming a previous RGB‐based study.

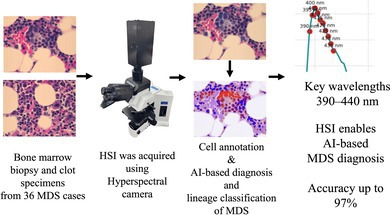

## 1 Introduction

The use of artificial intelligence (AI) has become widespread in recent years, driven by advances in computing technology over the past several decades that have accelerated its development. In medicine, AI is increasingly being used to assist with diagnostics in radiology and dermatology [1, 2]. In particular, advances in image analysis technology have enhanced diagnostic pathology, enabling the digitalization of glass slides, such as through whole‐slide imaging. These developments have facilitated the development of automated pathological diagnostic methods [3].

Several studies have attempted to automate pathological diagnosis by training deep convolutional neural networks (DCNNs) on large datasets of specimen images [4]. However, DCNNs are plagued by several problems. One major issue is the need for a large number of parameters, posing challenges for pathologists in interpreting the learning mechanisms and explaining the rationale for AI‐based diagnoses. This lack of explainability impedes its broader integration into clinical practice [5]. Another disadvantage of DCNNs is the need for large datasets of case images. Even simple image classification requires more than 1000 images, whereas achieving a highly accurate diagnosis will need more than 10 000 images. Consequently, research on DCNN‐based AI diagnosis is often restricted to large hospitals that can acquire sufficiently large numbers of specimens [6].

Spectral imaging, first introduced by Goetz in the 1980s, integrates conventional imaging and spectroscopy to obtain spatial and spectral information of an object. It was initially developed for remote sensing applications, such as satellite imaging, and was later extended to biomedical engineering. A hyperspectral camera (HSC) is a visual and near‐infrared (VNIR) sensor camera capable of detecting electromagnetic radiation emitted by objects with high spectral resolution. In general, images are three‐dimensional, composed of the spatial two‐dimensional XY plane and color components in the Z direction. Most conventional cameras produce RGB images containing only three color bands (red, green, and blue). RGB‐based imaging methods have known limitations in the early detection and identification of tissue abnormalities. In contrast, hyperspectral imaging (HSI) acquires a three‐dimensional data cube comprising two spatial dimensions and one spectral dimension, consisting of hundreds of continuous spectral bands that provide a spectral fingerprint for each pixel. Biomedical HSI can take advantage of the spatial relationships among neighboring spectra, enabling more elaborate spectral‐spatial models for improved classification. One important advantage of HSI is its ability to detect biochemical and morphological changes, facilitating early disease diagnosis by capturing reflectance or absorption features that cannot be identified in conventional RGB images. Therefore, in vivo, HSI enables non‐invasive observation of metabolic processes and disease‐related changes in living organisms, thereby enhancing biological understanding of diseases [7, 8, 9, 10]. Several approaches have been successfully applied to biomedical imaging, including whiskbroom, pushbroom, staring, and snapshot. The whiskbroom and pushbroom systems offer wide wavelength ranges and high spectral resolution. However, they require long image acquisition times and complex hardware configurations, making them primarily suitable for microscopic applications, where acquisition time is not a concern. The staring mode can be assembled compactly and easily integrated with other instruments while maintaining relatively high spectral resolution. Staring systems are well suited for analyzing organs, tissues, and cells, as they can be coupled with cameras, endoscopes, or microscopes. However, they are more expensive than the others. The snapshot rapidly acquires the spectral data cube, making it suitable for studies involving endoscopy or oxygen saturation measurements. However, its spectral and spatial resolution are limited. In summary, each mode has its own advantages and disadvantages in biomedical analysis, and the appropriate choice depends on the specific objectives of the study.

HSI has been clinically applied across a wide range of areas, including carcinoma detection, early diagnosis of diabetic skin complications, fundus and retina imaging analysis for diagnosing neurodegenerative diseases, and mapping blood oxygen saturation in various wound types [10, 11, 12, 13, 14, 15]. HSI has also been used in fluorescence detection, such as spectral karyotyping based on fluorescence in situ hybridization [16].

In histopathology, HSI provides a principled basis for tissue discrimination, as biochemical composition directly governs light absorption and reflectance. Consequently, tissues with distinct biochemical compositions, such as varying levels of hemoglobin and nucleic acids, exhibit characteristic spectral signatures [17]. HSI enables objective and quantitative tissue characterization, surpassing the qualitative results of traditional methods [18]. Previous studies suggest that HSI has broad potential applications, including detecting diverse lesions (carcinoma, melanoma, and non‐neoplastic lesions), discriminating color‐stained objects, quantitatively assessing the uptake of histological stains, identifying emission on tissue microarrays, evaluating the protective effect of erythropoietin on diabetic retinal cells, facilitating automated white blood cell segmentation, and, notably, extracting sufficient diagnostic information even from unstained pathological specimens for early diagnosis [17, 19, 20, 21, 22, 23, 24, 25].

Furthermore, several studies have demonstrated the potential of HSI to visualize molecular changes associated with tumor progression, including C6 glioma and Gleason 7 prostatic acinar adenocarcinoma progressing to metastasis [26, 27]. In summary, HSI facilitates early diagnosis and prognostic evaluation, offering improvements in morphological and molecular pathological diagnosis [7].

HSI offers a distinct advantage in pathological studies. As noted earlier, DCNNs typically require volumes of RGB pathological image data. In contrast, HSI can acquire rich color data across multiple wavelengths, enabling the extraction of sufficient color information from relatively smaller datasets for more accurate pathological diagnosis. These attributes motivated our team to evaluate the diagnostic utility of AI in rare neoplasms, where accumulating enormous data on specimens is difficult [28].

Previous studies have established HSI as an objective method for diagnostic pathology in many organs [6, 14, 28, 29, 30, 31, 32, 33]. Recent reviews have emphasized the significance of integrating HSI in machine learning and AI for precision diagnosis and standardized clinical workflows, although technical complexity and the need for specialized training remain barriers to broader clinical adoption [14, 34].

Consequently, the development of diagnostic pathological AI systems trained on HSI remains an active area of research. Several studies investigated the utility of AI in diagnosing myelodysplastic syndrome (MDS) using bone marrow smear and flow cytometry data [35, 36, 37]. MDS is a clonal hematopoietic disorder characterized by dysplastic bone marrow morphology and risk of transformation to acute myeloid leukemia (AML). Histopathological diagnosis of MDS relies on identifying subtle cytological dysplasia, a task that requires considerable expertise. Moreover, MDS is relatively rare, causing difficulty in accumulating the large datasets required for DCNN‐based AI systems [38]. Recently, Ishijima et al. [39] developed AI models and evaluated their accuracy in diagnosing MDS from biopsy specimens by combining two originally developed models.

To our knowledge, no prior reports have investigated HSI‐based histopathological diagnosis of bone marrow. Unlike previous studies that relied on RGB imaging, our approach leverages the full spectral fingerprint of each pixel. This enables the discrimination of subtle biochemical differences in dysplastic cells that are invisible to conventional imaging, thereby achieving higher accuracy. Furthermore, this is the first report to evaluate the diagnostic utility of AI trained on HSI data from bone marrow specimens.

## 2 Materials and Methods

### 2.1 Classification Based on the Lineage of Hematopoietic Cells on Pathological Specimens

This study analyzed data from 3442 patients who underwent bone marrow aspiration and biopsy at Showa Medical University Fujigaoka Hospital from 2015 to 2023. Thirty‐six patients were diagnosed with MDS based on the World Health Organization classification, fifth edition [40]. Specimens were collected from these patients and classified into three groups according to the dysplastic hematopoietic cell lineage classification described by Matsuda et al. [41].

**FIGURE 1:**
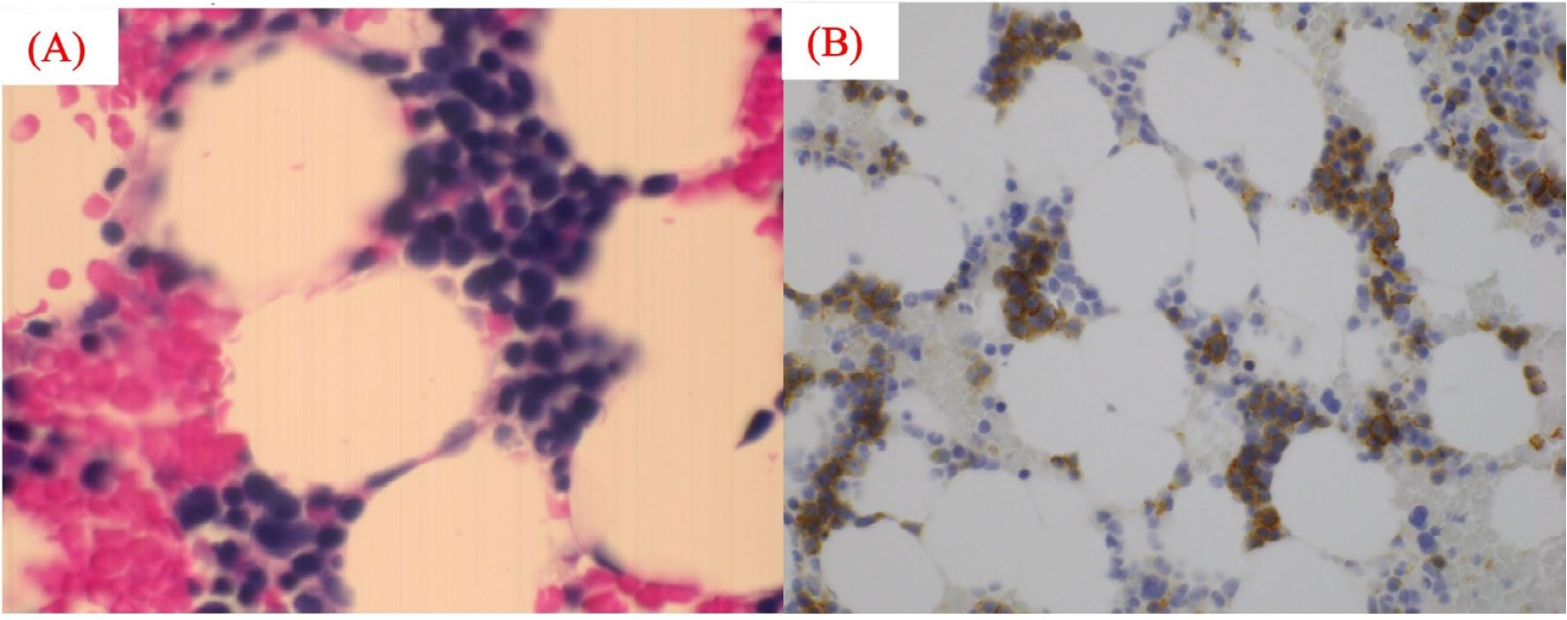
Representative histological images from group A (40× magnification). (A) Histological image of single‐lineage or multilineage myelodysplastic syndrome specimens showing dysplastic erythroid cells on hematoxylin and eosin staining. (B) Dysplastic erythroid cells positive for CD71.

**FIGURE 2:**
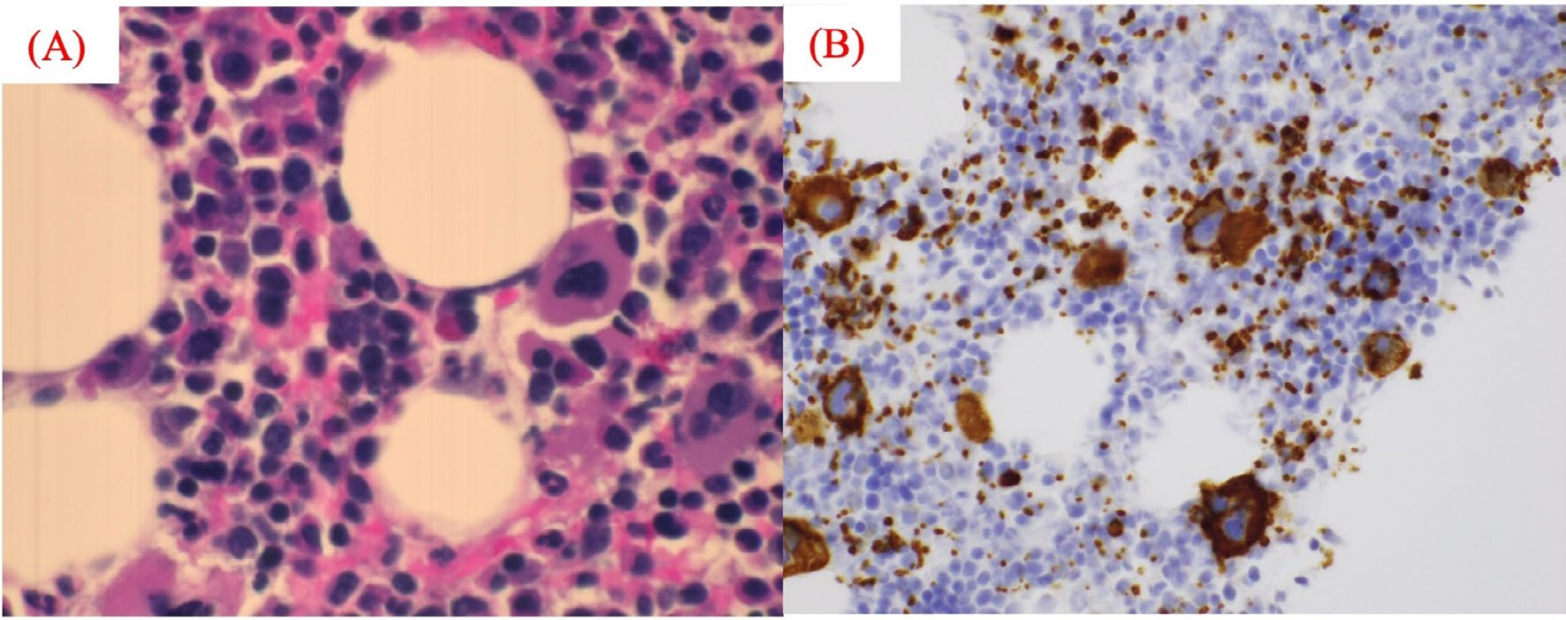
Representative histological images from group B (40× magnification). (A) Histological image of single‐lineage or multilineage myelodysplastic syndrome specimens showing dysplastic megakaryocytes on hematoxylin and eosin staining. (B) Dysplastic megakaryocytes positive for CD42b.

**FIGURE 3:**
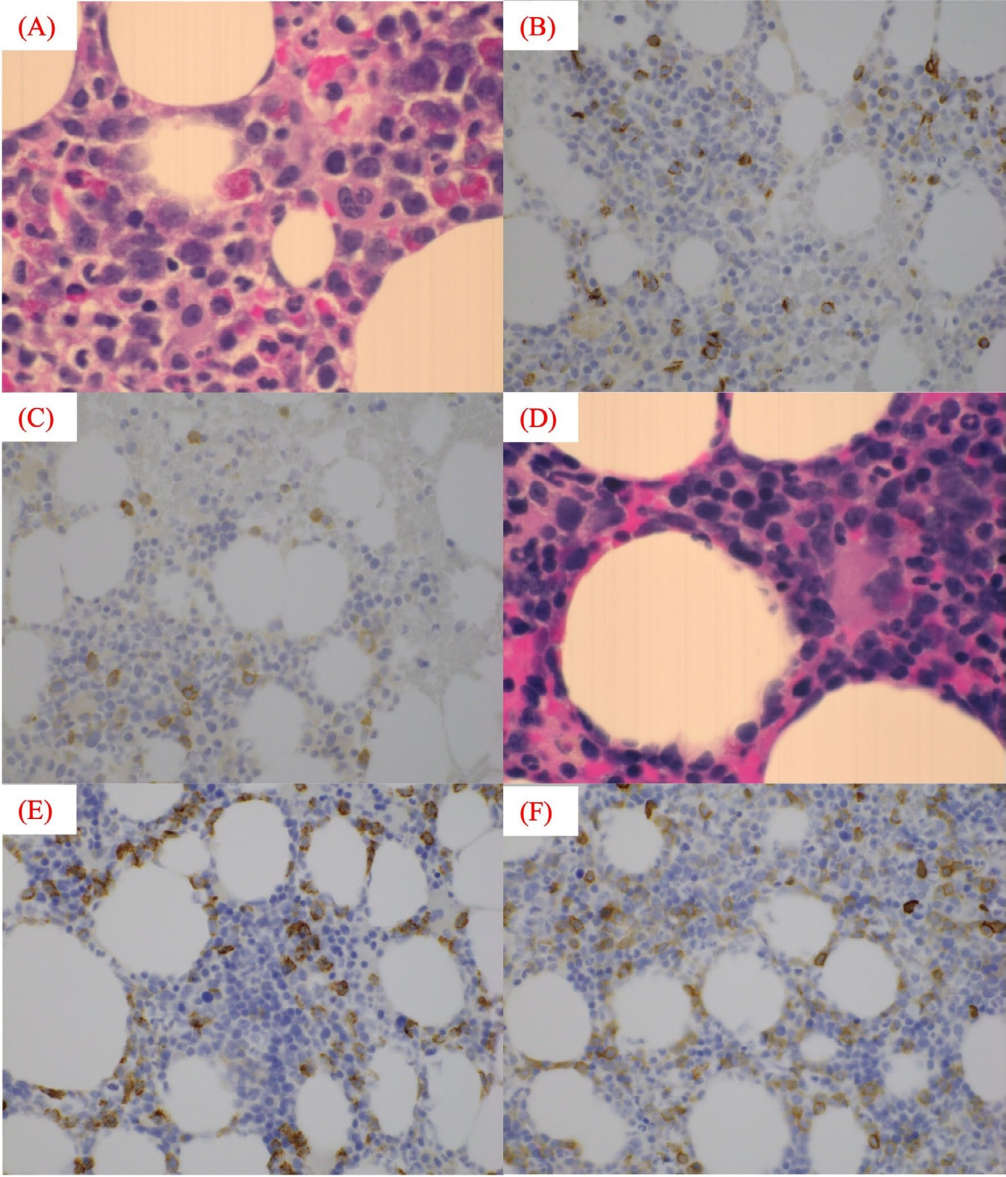
Representative histological images from group C (40× magnification). (A) Histological image of myelodysplastic syndrome with increased blasts level 1 (MDS‐IB1) on hematoxylin and eosin (H&E) staining. (B) A mild increase in blast cells, indicating CD34 positivity. (C) A mild increase in blast cells, indicating C‐KIT positivity. (D) Histological image of MDS‐IB2 on H&E staining. (E) Moderate‐to‐severe increase in blast cells, indicating CD34 positivity. (F) Moderate‐to‐severe increase in blast cells, indicating C‐KIT positivity.

**FIGURE 4:**
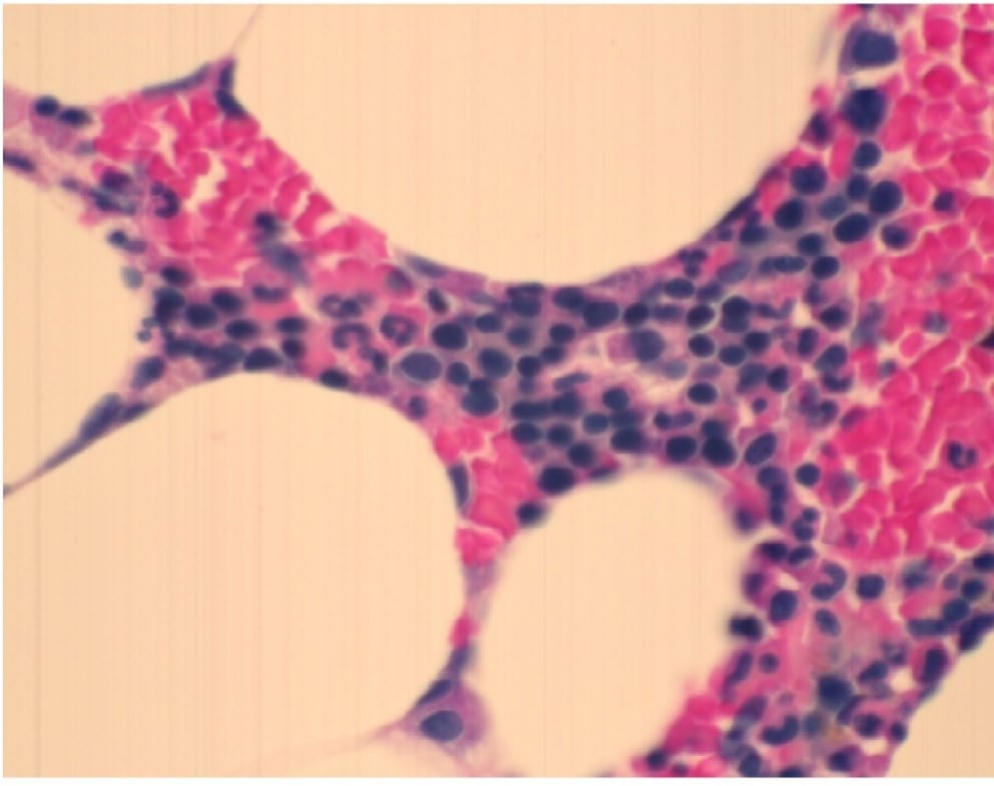
Representative histological image from group D showing normal erythroblasts (40× magnification).

**FIGURE 5:**
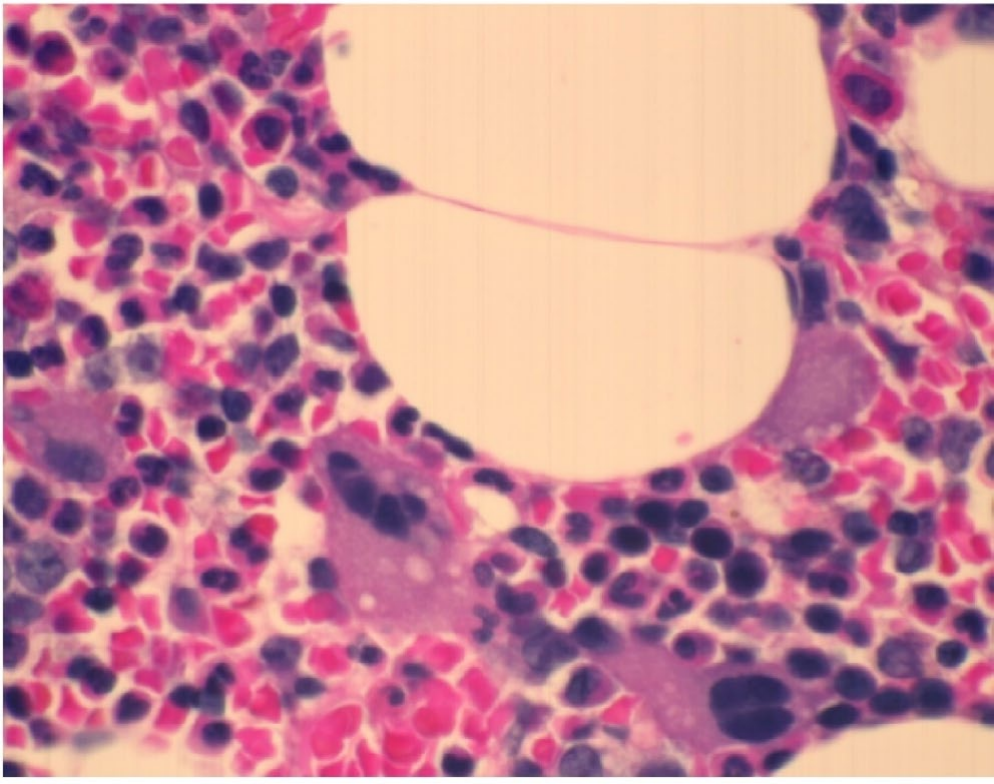
Representative histological image of group E containing normal megakaryocytes (40× magnification).

**FIGURE 6:**
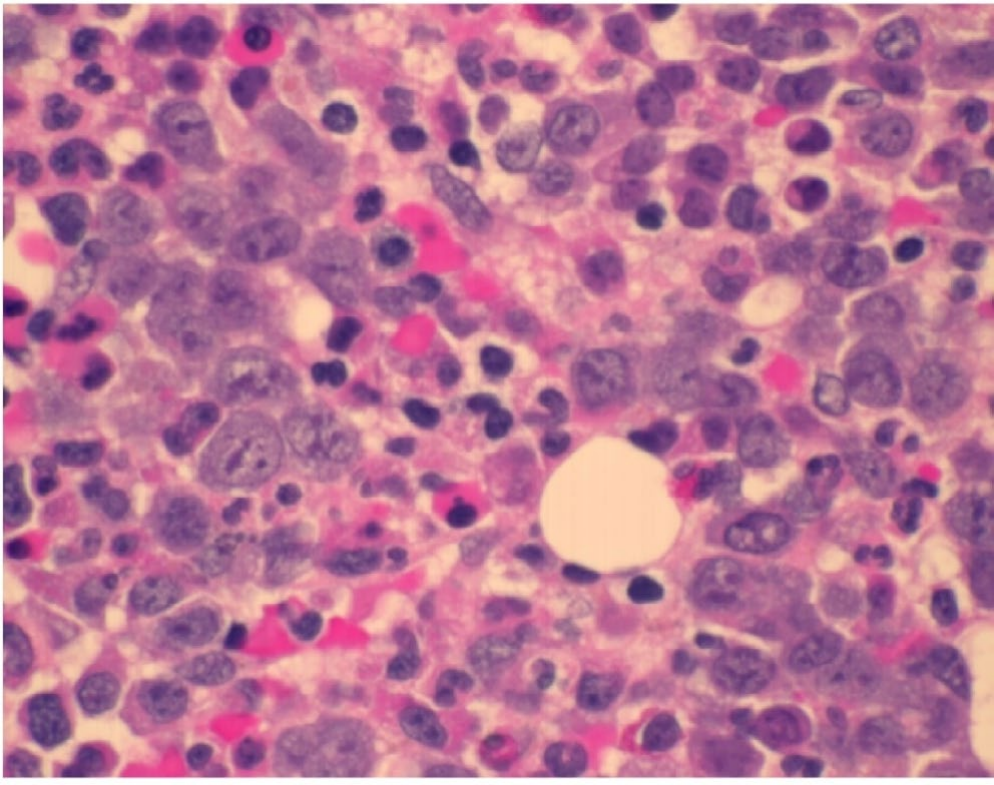
Representative histological image of group F, which was diagnosed with megaloblastic anemia (40× magnification).

Two pathologists (Y.U. and T.O.) histologically evaluated the specimens of myelodysplastic cells. Among specimens from MDS cases diagnosed with single‐lineage (SLD) or multilineage (MLD) dysplasia, those containing erythroid dysplastic cells were classified as group A (Figure 1), and those containing megakaryocytic dysplastic cells were classified as group B (Figure 2). The cases diagnosed with MDS‐IB were further classified as increased blasts 1 (IB1) or increased blasts 2 (IB2) and placed into group C (Figure 3). The control groups consisted of randomly selected normal bone marrow specimens and were designated as groups D and E. For group D, normal erythroblasts were annotated (Figure 4), while for group E, normal megakaryocytes were annotated (Figure 5). Furthermore, we randomly selected specimens diagnosed with megaloblastic anemia (MA), which can be histomorphologically difficult to distinguish from MDS, and classified them as group F (Figure 6). In each group, pathologists marked hematoxylin–eosin slides at one or two locations per specimen for the target cells: dysplastic hematopoietic cells in groups A and B; blast cells in group C; normal erythroblasts and megakaryocytes in groups D and E, respectively; and megaloblasts in group F.

### 2.2 Hardware of the HSI System

We used an HSC1702 HSC (Iris Inc., Japan), which employs a pushbroom scanning technique, has a measurement wavelength range of 350–1050 nm, a wavelength resolution of 5 nm, 141 intensity bands (in the Z direction), and a spatial resolution of 640 × 480 pixels. HSIs were acquired using a charge‐coupled device sensor. Total acquisition time was 16 s per photo.

### 2.3 Assessing the Diagnostic Accuracy of Machine Learning With HSI

**FIGURE 7:**
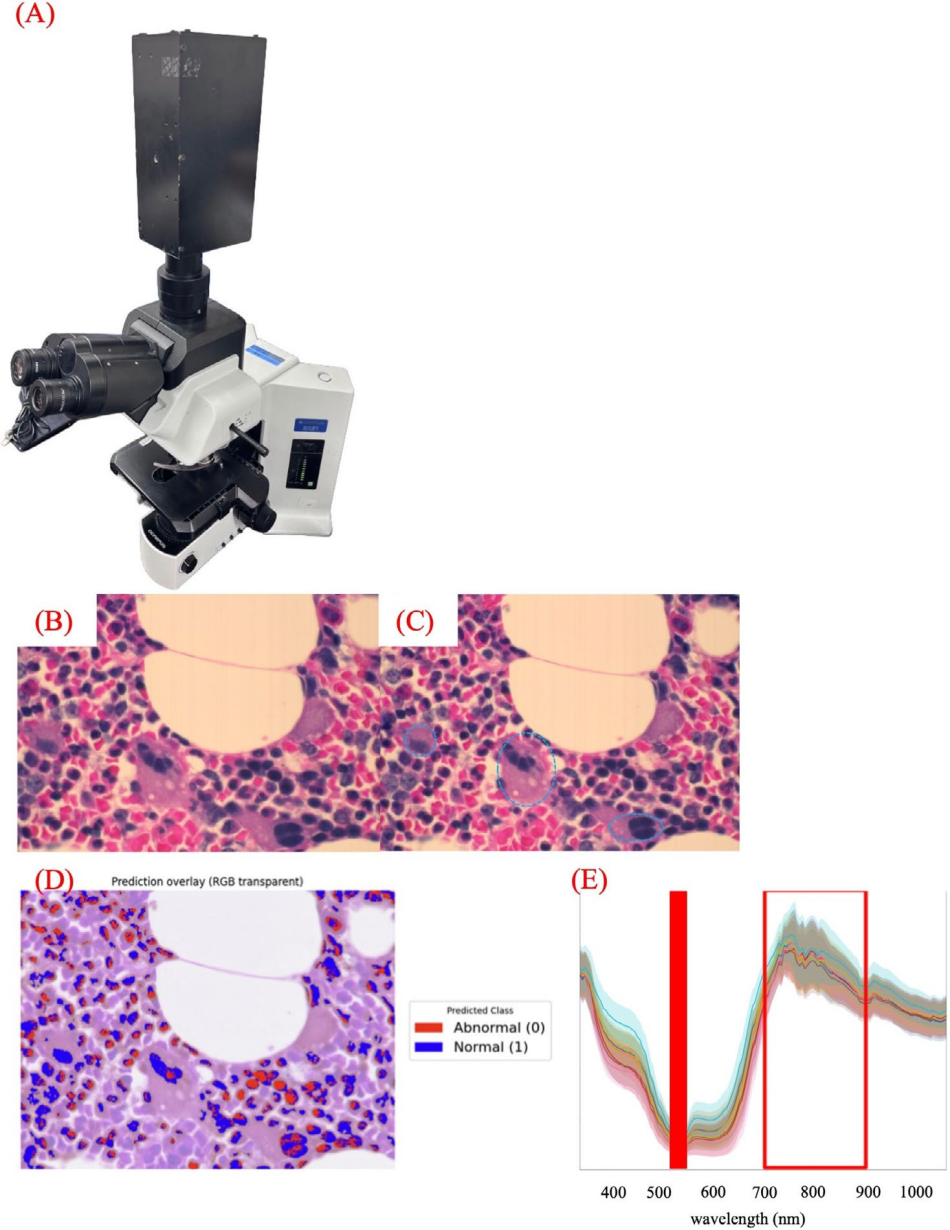
Creation of the prediction algorithm. (A) Target cells were captured on each specimen using a hyperspectral camera. (B) False‐color RGB images were generated for display on a standard monitor. (C) Target cells indicated by blue circles were annotated as the region of interest (ROI). (D) Target (blue) or non‐target cells (red) in the ROI predicted by artificial intelligence. (E) Spectral information of target cell nuclei was extracted. The wavelengths of 540 nm (red band) and 700–900 nm (red square) were significant.

The HSC was directly connected to the eyepiece lens of an Olympus microscope, and HSIs were captured using a white light‐emitting diode bulb as the light source (Figure 7A). Magnification was 10× for the eyepiece lens and 40× for the objective lens. Myelodysplastic cells were selected by lineage from the cell clusters on HSI, yielding the following: 348 dysplastic erythroblasts from 7 specimens in group A; 58 micromegakaryocytes from 14 specimens and 38 isolated megakaryocytes from 14 specimens in group B; 268 blast cells from 12 specimens in group C; 95 normal erythroblasts from 2 specimens in group D; 6 megakaryocytes from 3 specimens in group E; and 155 megaloblasts from 5 specimens in group F. Because they contain 141 spectral bands, HSIs cannot be visualized on a standard display. Initially, only RGB color components could be extracted, generating false‐color RGB images (Figure 7B). Next, target cells were annotated in a circular region of interest (ROI) on the RGB images (Figure 7C), after which an AI model predicted the target (blue) and non‐target (red) cells in the ROI (Figure 7D). The RGB images were divided into rough pixel clusters with multiple adjacent pixels of similar color constituting a single cluster. The hyperspectral data for each pixel population were averaged to identify significant wavelengths at 540 nm (red band) and 700–900 nm (red square) (Figure 7E). Next, the dataset was split at the sample level into training and evaluation sets. Training data were collected from multiple cells across different samples. The evaluation sets consisted of samples completely unseen by the model and were never used during training, ensuring that no pixels from the same sample were shared between training and evaluation sets. Furthermore, the training set was divided into 80% training and 20% test subsets using a stratified sample‐level split, and cross‐validated by repeating the split five times. Furthermore, the data extracted from the cell nucleus were divided into training and test sets at the pixel level, with different proportions for each analysis, as described subsequently. For classes with fewer samples, a larger proportion of the data was allocated to the training set to ensure that the model could learn sufficient spectral features and capture intra‐class variability.

To develop a model for facilitating MDS diagnosis, we evaluated its diagnostic accuracy across six key dimensions using machine learning: (1) myelodysplastic and blast cells versus normal hematopoietic cells; (2) group F megaloblasts versus normal erythroids/megakaryocytes; (3) normal erythroid cells versus normal megakaryocytes; (4) dysplastic erythroblasts + blast cells versus dysplastic megakaryocytes; (5) dysplastic erythroblasts versus blast cells; and (6) micromegakaryocytes (category A) versus multinucleated megakaryocytes (category B) [38, 40, 41]. We used different machine learning models for each classification task, and the hyperparameters were optimized using grid search.

### 2.4 Visualization of the Hyperspectral Data by Principal Component Analysis

Principal component analysis (PCA) was performed to minimize redundancy and capture dominant spectral variations. We averaged the hyperspectral data extracted from the cell nucleus and compressed the color data from 141 dimensions into principal components for visualization.

## 3 Results

### 3.1 Clinicopathological Features of the Patients in Each Group

Of the 3442 patients who underwent bone marrow tests, 36 were diagnosed with MDS. Three patients were excluded because we could not obtain sufficient cells for evaluation in a single field of view under a 40× objective lens. The remaining 33 patients were then classified into groups A (*n* = 7), B (*n* = 14), and C (*n* = 12). Five patients were randomly selected as normal bone marrow controls, two of whom were classified in group D, and the remaining three in group E. Furthermore, we randomly selected five specimens diagnosed with MA and classified them in group F.

The average age of patients with MDS classified into groups A–C was 75.91 ± 10.35 years (median: 76; range: 59–97; male: female ratio = 20:13; *N* = 33), which is close to the age and sex of onset described in the WHO classification fifth edition (median age 77, slightly predominant male). The average age from groups A to C was 77.29 ± 10.3, 74.6 ± 10.44, and 76.58 ± 10.07 years, respectively. In the control group, the average age was 69 ± 12.86 years in the patients with MA (group F), which also corresponded to the onset age in the WHO fifth classification [40]. Genetic analytical data were available for 19 patients, 11 of whom had a *WT1* mutation. Chromosomal abnormalities were identified in 18 cases: del(5) or del(5q) in eight, del(7) or del(7q) in seven, and del(20q) in four. The clinicopathological data for the MDS and control groups are summarized in Table 1, and the genetic mutations are summarized in Table 2.

**TABLE 1:** Clinicopathological data of patients in the MDS and control groups.

Group	Patient data					Pathological diagnosis			
	Number	Male	Female	Average age	SD	SLD	MLD	IB1	IB2
A	7	3	4	77.29	10.3	2	5	0	0
B	14	8	6	74.64	10.44	12	2	0	0
C	12	9	3	76.58	10.07	0	0	5	7
A + B + C	33	20	13	75.91	10.35	14	7	5	7
D	2	1	1	63	14.14				
E	3	1	2	65.67	13.87				
D + E	5	2	3	64.6	12.18				
F	5	3	2	69	12.86				

Abbreviations: IB, increased blasts; MDS, myelodysplastic syndromes; MLD, multilineage dysplasia; SD, standard deviation; SLD, single‐lineage dysplasia.

**TABLE 2:** Genetic mutations in 19 reported cases.

Group	Patients	*WT1*	*CSF1R*	*EGR1*	Negative
A	7	2	2	2	1
B	14	7	0	0	5
C	12	2	0	0	0
A + B + C	33	11	2	2	6

*Note:* In group A, two patients exhibited both *CSF1R* and *EGR1* mutations.

### 3.2 Evaluation of the Diagnostic Accuracy of Using Machine Learning on HSI

**FIGURE 8:**
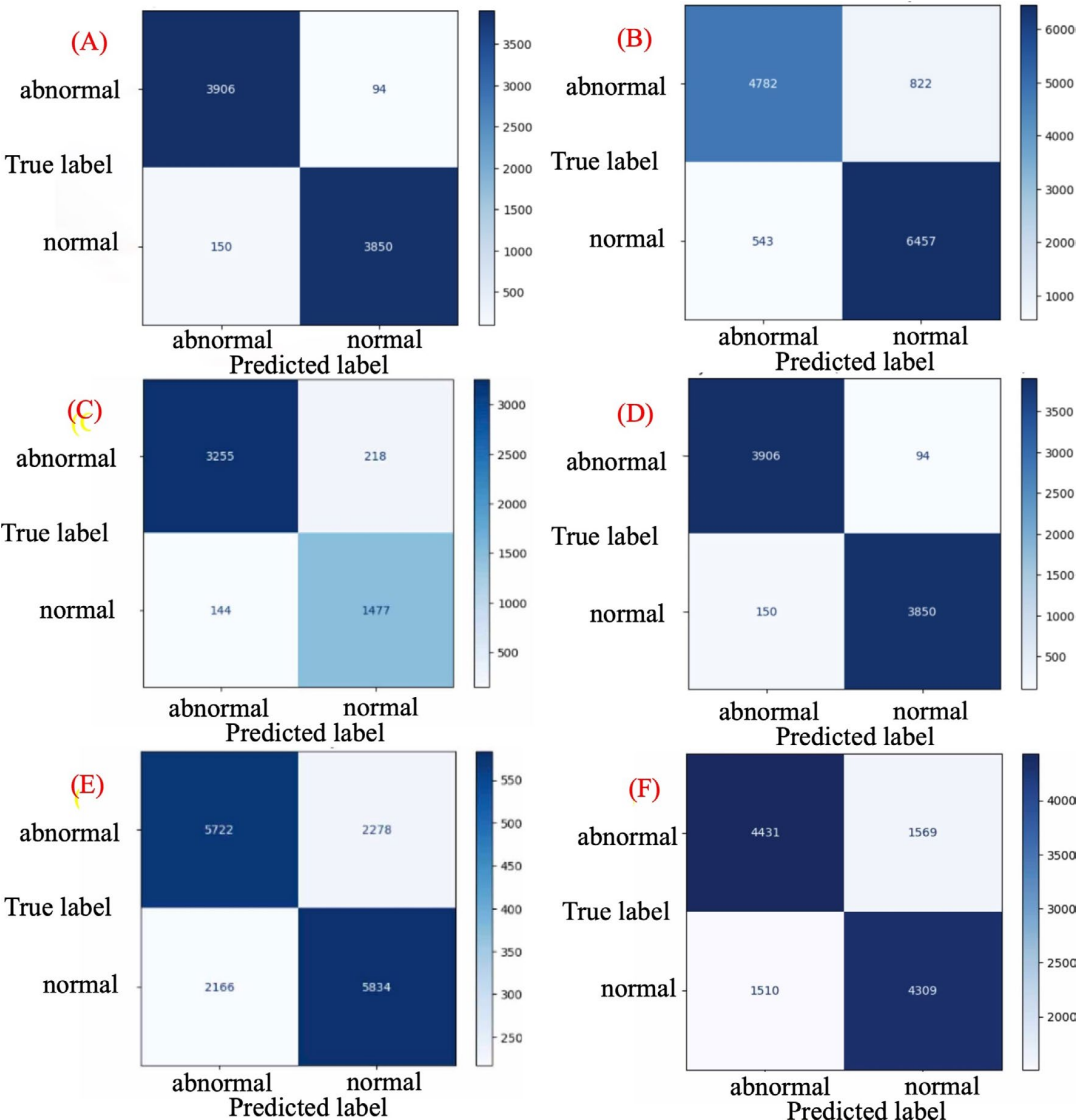
Evaluation of test data performance. The accuracy for the test data was (A) 97%, (B) 89%, (C) 93%, (D) 72%, (E) 82%, and (F) 74%.

1. Dysplastic cells (groups A–C) versus normal hematopoietic cells (groups D and E) + megaloblasts (MA; group F) (Figure 8A).
  The data extracted from groups A–C were divided into training and test sets at a 7.25:1 ratio, while those from groups D–F were divided at a 4:1 ratio. As a machine learning model, multilayer perception (MLP) was used. The diagnostic accuracy for the training data was 90%, whereas that for the test data was 97%.
2. Normal erythroblasts (group D) + normal megakaryocytes (group E) versus megaloblasts (group F) (Figure 8B)
  The data extracted from groups D and E were divided into training and test sets at a 1.5:1 ratio, while those from group F were divided at a 4:1 ratio. Random forest was applied only for this evaluation because it performed better than MLP. The accuracy for the training data was 91% and that for the test data was 89%.
3. Normal erythroblasts (group D) versus normal megakaryocytes (group E) (Figure 8C).
  The data extracted from group D were divided into training and test sets at a 1:1 ratio, while those from group E were divided at a 2:1 ratio. MLP was used for machine learning. The accuracy for the training data was 65%, while that for the test data was 93%.
4. Dysplastic erythroblast cells (group A) + blast cells (group C) versus dysplastic megakaryocytes (group B) (Figure 8D).
  The data extracted from groups A and C were divided into training and test sets at a 8.5:1 ratio, while those from group B were divided at a 1.5:1 ratio. MLP was used as the machine learning model. The accuracy for the training data was 57%, while that for the test data was 72%.
5. Dysplastic erythroblast cells (group A) versus blast cells (group C) (Figure 8D).
  The data extracted from group A were divided into training and test sets at a 6:1 ratio, whereas those from group C were divided at an 11:1 ratio. As a machine learning model, MLP was used. The accuracy for the training data was < 50%, while that for the test data was 82%.
6. Dysplastic multinucleated megakaryocytes versus micromegakaryocytes (Figure 8F).
  The data extracted from multinucleated megakaryocytes were divided into training and test sets at a 13:1 ratio, whereas those from micromegakaryocytes were divided at a 10:1 ratio. MLP was also used for machine learning. The accuracy for the training data was < 50%, while that for the test data was 74%.

The accuracies and machine learning models for each evaluation are summarized in Table 3.

**TABLE 3:** Diagnostic and discriminative accuracy.

Evaluation no.	Group	Discrimination	Machine learning model	Accuracy (%)
1	A + B + C vs. D + E + F	MDS vs. normal bone marrow/MA	MLP	97
2	D + E vs. F	Normal erythroblasts + megakaryoblasts vs. megaloblasts	Random Forest	89
3	D vs. E	Normal erythroblasts vs. normal megakaryoblasts	MLP	93
4	A + C vs. B	Dysplastic erythroblasts+ blast cells vs. dysplastic megakaryoblast	MLP	72
5	A vs. C	Dysplastic erythroblasts vs. blast cells	MLP	82
6	Category A vs. category B (in group B)	Dysplastic megakaryoblasts with multiple widely separated nuclei vs. micromegakaryoblasts	MLP	74

Abbreviations: MA, megaloblastic anemia; MDS, myelodysplastic syndrome; MLP, multilayer perception.

### 3.3 Evaluation of the Wavelength Contributing to Target Cell Detection

In the detection of dysplastic cells with altered chromatin organization, the most significant wavelengths ranged 390–440 nm, corresponding to the violet/near‐ultraviolet (UV) region, where chromatin structures and nucleic acid‐related optical properties exhibit stronger absorption and scattering.

**FIGURE 9:**
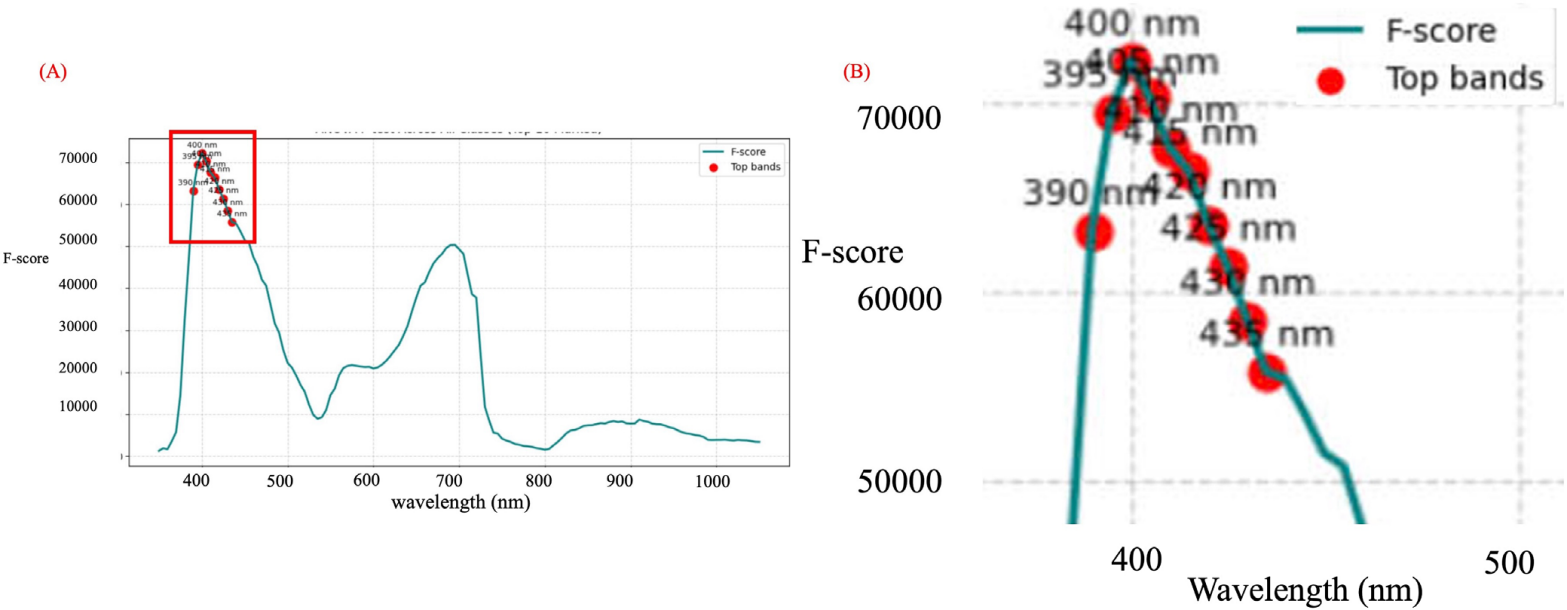
Evaluation of the wavelength contributing to target cell detection. (A) The wavelength range of 390–440 nm was significant in the detection of dysplastic cells (area in red square). (B) An enlarged view of the region in the red square.

Figure 9B presents a magnified view of the area inside the red box in Figure 9A.

### 3.4 Analysis of the Main Components Contributing to the Detection of MDS Cells

The quantity of the first principal component (PC1) differed between group F (megaloblasts were the highest in PC1) and groups A and C (dysplastic erythroblast cells and blast cells were the lowest in PC1).

**FIGURE 10:**
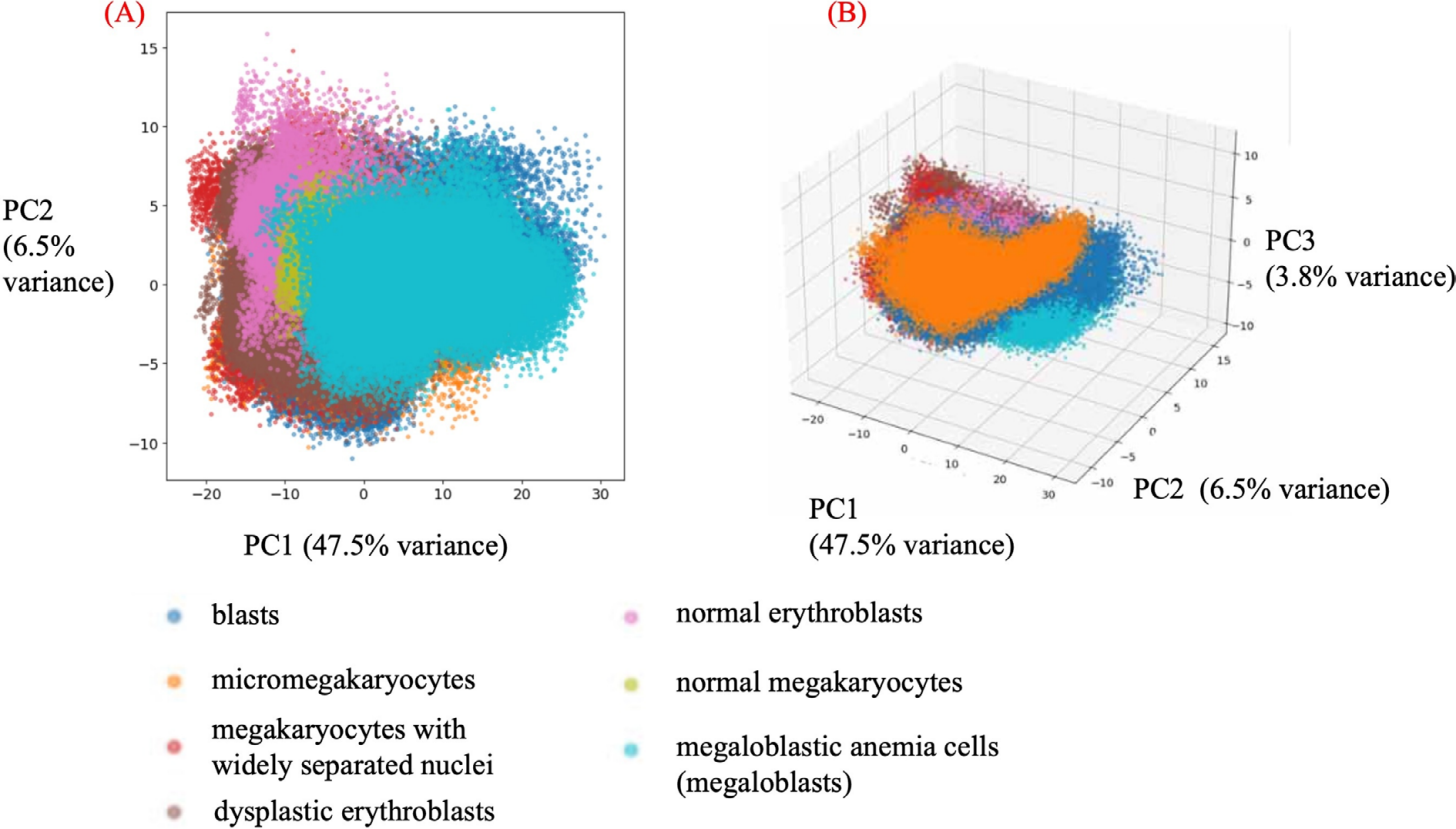
Analysis of the main components contributing to the detection of myelodysplastic cells. (A) The quantity of the first principal component (PC1) differed between group F (megaloblasts were the highest in PC1) and groups A and C (dysplastic erythroblast cells and blast cells were the lowest in PC1). (B) Three dimensions of the principal component analysis plots.

Figure 10 presents the two‐dimensional and three‐dimensional PCA plots.

## 4 Discussion

MDS is characterized by clonal neoplastic proliferation of hematopoietic stem cells, dysplastic hematopoietic cells in a single or multiple lineages, ineffective hematopoiesis, genetic mutations, and the potential for frequent transformation into AML [38]. Diagnosis of MDS, a preleukemic disorder, requires a comprehensive evaluation of clinical findings, laboratory data, and bone marrow morphology. Pathological examination is essential in establishing definitive diagnostic criteria for MDS, including the blast cell ratio, number of lineages, and dysplastic cell‐to‐nucleated cell ratio (> 10%). Transformation to AML occurs in approximately 30% of patients with MDS. Therefore, accurate diagnosis of MDS is essential for prognosis; however, early diagnosis is sometimes difficult [42]. A major contributing factor is the global shortage of diagnostic pathologists, especially specialists trained in bone marrow pathology. Consequently, not all hospitals provide sufficient pathological evaluation for patients with MDS [43].

AI‐based pathology diagnostic systems are expected to alleviate the shortage of pathologists and enable rapid diagnosis and treatment. Several attempts have been made to develop AI systems (Table 4) for bone marrow pathology [12, 14, 15, 21, 22, 23, 24, 25]. However, issues persist in the development of AI pathology diagnostic systems. One major problem is the need for large volumes of pathological data and a sufficient number of specimens in developing reliable AI‐based pathological diagnostic systems. Therefore, the research on AI‐based pathological diagnostic systems has been limited to large medical organizations [28].

**TABLE 4:** Clinicopathological study evaluating AI for MDS diagnosis on specimens.

No.	Year	Author	Aim	Material	Sensitivity, specificity, or accuracy
1	2019	Kimura [21]	Automated diagnostic systems	PBS	93%–99.8% 96%–100%
2	2020	Mori [12]	Assessments of predicting decreased granules	BMS	85.2% 98.9%
3	2020	Wu [22]	Deep learning models to facilitate diagnosis	BMS	67.40%
4	2021	Bruck [23]	Prediction for genetic mutations	BMS	
5	2022	Lee [14]	Identification of hematopoietic lineage and detection of dysplastic cells	BMA	90%–99.9%
6	2022	Wang [24]	Establishment of AI for identifying AA, MDS, and AML	BMS	92.90%
7	2025	Romano [25]	Developments of AI for the MDS diagnosis	PBS	95%, 94%
8	2025	Ishijima [15]	Establishment of Artificial intelligence model for classifying myeloid diseases	BMB	76.90%
9		Our study	Establishment of AI, diagnose and classify MDS	BMA + BMB	97% (MDS or not)

Abbreviations: AA, anaplastic anemia; AI, artificial intelligence; AML, acute myeloid leukemia; BMA, bone marrow aspiration; BMB, bone marrow biopsy; BMS, bone marrow smear; MDS, myelodysplastic syndrome; PBS, peripheral blood smear.

An HSC contains a VNIR sensor with high spectral resolution and more than 100 spectral bands. In diagnostic pathology, HSI is a non‐invasive method that can recognize and discriminate histological features that are otherwise undetectable on pathological RGB images [44]. HSI can capture spectral information useful in pathological diagnosis from a relatively small number of specimens. Ohike et al. reported high diagnostic accuracy of AI systems on HSI for distinguishing grade 1–2 neuroendocrine tumors from neuroendocrine carcinomas, using a small cohort of 13 cases [28].

After obtaining the HSIs, the dataset was split into training and evaluation sets at the sample level and cross‐validated to evaluate the generalizability and reduce the risk of overfitting. Training data were collected from multiple cells across different samples. After training, testing was performed using evaluation sets consisting of completely unseen samples rather than pixels from the same cells used during training, ensuring a realistic assessment of generalization. In evaluation 5, the accuracy on the training data was low (< 50%), suggesting that the model was not simply overfitting to spatially correlated pixels.

First, the diagnostic accuracy for distinguishing MDS cells (groups A–C) from non‐MDS cells (groups D–F) was evaluated using bone marrow biopsy and aspiration specimens. Previous AI‐based approaches for bone marrow pathology have primarily relied on RGB images or flow cytometry data [35, 36, 37]. While Ishijima et al. [39] previously achieved 67.9% diagnostic accuracy for MDS using RGB image‐based AI systems in 53 MDS cases, our HSI‐based approach achieved up to 97% accuracy with only 33 cases, suggesting that the rich spectral information provided by HSI can compensate for smaller sample sizes. HSI‐based AI systems may help differentiate MA from MDS. MA is a hematopoietic disease characterized by a disorder of DNA synthesis caused by Vitamin B12 deficiency, presenting clinically with fatigue or shortness of breath and elevated mean corpuscular volume on blood tests. A diagnosis of MA requires a comprehensive evaluation based on clinical information and blood and bone marrow tests. A clear distinction between MDS and MA is important because of marked differences in clinical course, treatment, prognosis, and patient outcomes. However, distinguishing between MDS and MA based on pathological specimens can be difficult, with a diagnostic accuracy of 55%–84% [45, 46]. Our AI systems achieved high accuracy (97%) in discriminating MDS from control groups, including MA, which was attributed to differences in principal components between MA (group F) and the MDS subgroups (groups A and C). Also, the diagnostic accuracy achieved for distinguishing between normal erythroblasts or megakaryocytes and MA was 89%. These findings indicate the utility of our AI diagnostic systems in distinguishing between MA and MDS and in diagnosing MA, particularly in hospitals lacking expert hematologists or pathologists.

The discrimination accuracy between normal erythroblasts and normal megakaryocytes reached 93%, indicating that the diagnostic AI systems developed in the present study may help confirm the nature of individual cells, especially in hospitals where immunostaining is unavailable. In distinguishing between different lineages of dysplastic cells, the accuracy was relatively high at approximately 82% between dysplastic erythroblasts and blast cells. Distinguishing between dysplastic erythroblasts (megaloblasts) and blast cells is very important for prognostic prediction, as overall survival and leukemia‐free survival in MDS‐IB1 and IB2 are significantly shorter than those in MDS‐SLD and MDS‐MLD [47]. However, identifying dysplastic erythroblasts and blast cells can be difficult, even for expert pathologists, due to a high nuclear‐to‐cytoplasmic (N/C) ratio and a similar chromatin pattern [48]. Further investigations into chromatin patterns in dysplastic cells and improvement of diagnostic accuracy would enhance the predictive accuracy of our AI systems, not only for use in local clinics without pathologists, but also in hospitals with full‐time pathologists. Although the accuracy in distinguishing normal megakaryocytes from normal erythroblasts was excellent (93%), the accuracy in distinguishing dysplastic erythroblasts and blast cells from dysplastic megakaryocytes was 72%, while that in distinguishing between multinucleated megakaryocytes and micromegakaryocytes was 74%, which are both relatively low. We presume that a lower accuracy is attributed to the morphologic diversity and complexity of dysplastic megakaryocytes, including nuclear morphology and intranuclear chromatin patterns, which are not observed in normal megakaryocytes. Furthermore, we assume that the reduced accuracy may have been influenced by interobserver variability among pathologists in distinguishing dysplastic cells of different lineages from blasts. Font et al. [49] found that the diagnostic concordance rate for MDS low blast (MDS‐LB) tends to be lower than that for MDS increasing blast (MDS‐IB), even among expert pathologists. Tanifuji et al. [50] reported that if a discordance occurs in the diagnosis between pathologists, the diagnostic accuracy of AI will also tends to be relatively low, indicating that the accuracy of machine learning‐based diagnostics may be influenced by diagnostic discordance, even between skillful pathologists.

PCA was employed in this study for three primary reasons. First, PCA reduces redundancy and captures dominant spectral variations. Second, it offers a more objective and reproducible approach to detecting cytomorphological differences than conventional RGB‐based evaluation, as it minimizes reliance on manual visual assessment and facilitates automated computer‐aided diagnosis. Third, recent evidence suggests that spectral decomposition via PCA preserves diagnostically relevant variance while diminishing computational demands, thereby enhancing the study by integrating quantitative spectral features and statistical robustness [34, 51, 52].

Although PCA is widely used for dimensionality reduction of HSI data, it has several limitations. PCA assumes linear relationships within the data, which may not adequately capture the complex nonlinear features inherent in biological tissues [53]. Furthermore, the principal components obtained are mathematical constructs that can be difficult to interpret in a biological or pathological context [54]. Finally, as an unsupervised method, PCA does not optimize for class discrimination, potentially limiting its performance compared with supervised approaches, such as linear discriminant analysis (LDA) [55].

Our findings showed distinct wavelength patterns between MA (group F) and MDS with dysplastic erythrocytes (group A) and MDS with increasing blast cells (group C), resulting in high accuracy in evaluation 1. An important wavelength range at which MDS can be distinguished from non‐MDS was the 390–440 nm range (UV), which corresponds to the violet‐near UV region, where chromophores relevant to dysplasia may contribute to enhanced absorption and scattering characteristics. Furthermore, endogenous fluorophores such as NADH and FAD, which indicate metabolic activity, emit in the 440‐ to 520‐nm range when excited near 390 nm, potentially providing contrast between metabolically active dysplastic cells and normal epithelium. At the morphological level, dysplastic nuclei exhibit increased size, altered chromatin condensation, and an elevated N/C ratio, all of which influence light scattering characteristics detectable within this wavelength range. Notably, direct DNA absorption peaks at 260 nm; therefore, the model's sensitivity at the 390‐ to 440‐nm range more likely reflects nucleic metabolic changes rather than direct nucleic acid absorption [56, 57].

Our study highlights several key advantages of applying HSI to diagnostic pathology. First, as described, we demonstrated the robust performance of machine learning and AI models trained on HSI data, even with a relatively small sample size. These findings indicate that HSI is useful for diagnostic pathology in bone marrow biopsy and could facilitate AI‐driven pathological research on MDS in settings where large‐scale data collection is challenging, such as community hospitals. Second, HSI‐based pathological diagnosis of MDS offers an objective approach that enables clear discrimination of MDS without requiring specialized experience, training, or additional immunostaining. Third, HSI‐based diagnosis is non‐invasive and label‐free. Lastly, the ability of HSI to detect spectral differences in color may help develop future applications for early diagnosis or molecular analysis. Our study is the first to evaluate the diagnostic utility of AI trained on HSI of bone marrow specimens. Although we employed established methods, our application of HSI to bone marrow diagnostic pathology represents a novel contribution to the field. However, HSI still has several limitations. First, algorithm optimization and guideline standardization remain insufficient. Second, most of the previous HSI‐based medical investigations, including ours, were conducted in a single center, which may be over‐adapted to the institution and lack generalizability [14]. Thirdly, high equipment costs and limited portability have constrained its widespread adoption. Furthermore, image acquisition time requires considerable time which may limit its immediate clinical applicability [58, 59].

Our research has several limitations. First, we evaluated whether the AI system could identify dysplastic cells within only a single lineage. However, MDS cases containing both dysplastic erythroblasts and dysplastic megakaryocytes were not evaluated. Second, HSI‐based quantitative evaluation of the number of dysplastic lineages, percentage of dysplastic cells (> 10%), or percentage of blast cells was not performed. Third, the ability of HSCs to detect genetic mutations in conditions such as MDS with chromosomal abnormalities, including del(5q), was not evaluated. Fourth, the specimens for control groups D and E were randomly selected from older patients with malignant lymphoma or solid neoplasms without invasion into the bone marrow; thus, the histological structure and genetic development of these specimens would differ significantly from those of specimens from young healthy people. Fifth, our study was conducted at a single institution, as in most previous studies. We performed strict data separation at the sample level to evaluate the model on completely unseen samples, and it showed reasonable performance, demonstrating a certain degree of generalization. However, the possibility remains that machine learning over‐adapted to staining and the microscope light source in our hospital, so we plan to accumulate sufficient cases in multiple institutions to improve generalization. Sixth, there was variation in the training/test data ratio across classes due to limited sample sizes. In future work, with more balanced datasets across classes, more standardized evaluation will be adopted using a sufficient number of cases.

In this study, our HSI‐based AI system achieved excellent accuracy in discriminating MDS from MA/normal controls. In a future investigation, we intend to evaluate the usefulness of our HSI‐based AI system in the diagnosis of other hematological diseases.

## 5 Conclusion

We evaluated the accuracy of AI‐based pathological diagnostic systems using HSI, achieving excellent diagnostic accuracy for several diagnostic points: MDS versus non‐MDS, normal erythroblasts versus normal megakaryocytes, and normal hematopoietic cells versus MA cells. These results demonstrate the utility of our AI system for diagnosing MDS and distinguishing it from other diseases. Furthermore, the accuracy of identification between dysplastic erythroblasts and blast cells was relatively high, indicating its utility in distinguishing MDS‐IB or MDS‐LB. In contrast, the accuracy of distinguishing between micromegakaryocytes and multinucleated megakaryocytes was relatively low. Overall, AI‐based diagnostic systems may be a useful and robust tool for diagnosing MDS by learning the diversity and complexity of dysplastic cells through the accumulation of sufficient sample sizes across multicenter institutions.

## Funding

The authors have nothing to report.

## Ethics Statement

This research was approved by Showa Medical University Research Ethics Review Board (Approve No. 2024‐029‐A).

## Consent

This study is a retrospective observational study; instead of obtaining direct informed consent from each patient, the research plan was published on the Showa Medical University website. Recruited participants were given the opportunity to opt out of their data being used.

## Conflicts of Interest

This study outsourced image analysis and image data management to Milk Inc., and the corresponding author doesn't manage any conflicts of interest.

## Data Availability

The data that support the findings of this study are available on request from the corresponding author. The data are not publicly available due to privacy or ethical restrictions.
